# Web-Based Augmented Reality vs. Interactive Presentation for Learning Caries Detection: A Randomized Study on Student Motivation

**DOI:** 10.3390/dj14010001

**Published:** 2025-12-19

**Authors:** Sofía Folguera, Carmen Llena, José Luis Sanz, Leopoldo Forner, María Melo

**Affiliations:** Department of Stomatology, Universitat de València, 46010 Valencia, Spain; sofia.folguera@uv.es (S.F.);

**Keywords:** Augmented Reality, dental education, motivation, caries detection

## Abstract

**Background/Objectives**: Augmented Reality (AR) is promising in dental education, yet its impact on *caries detection* training remains underexplored. This study aimed to compare the effect of a web-based AR (WebAR) learning object with a content- and interface-matched interactive 2D presentation on undergraduate students’ motivation to learn caries detection. **Methods**: Two learning objects were expressly designed using a real patient’s dental records: a WebAR image-tracking experience (built with Zapworks Studio^®^) and a 2D interactive presentation (built with Genially^®^). The WebAR object showed the patient’s 3D dental arches with tooth-level hotspots linking clinical and radiographic media. The 2D comparator mirrored the same assets and navigation, restricting visualization to 2D. Third-year dental students were randomly assigned to either the AR or Genially^®^ (G) group. After completing ICDAS-based caries identification, participants completed the 12-item Reduced Instructional Materials Motivation Survey (RIMMS) and provided open-ended feedback. Group differences were tested with the Mann–Whitney U test (*p* < 0.05). **Results**: Eighty-five students completed the study (AR n = 46; G n = 39). The AR group achieved a higher total RIMMS score (4.14 vs. 3.53 on a 5-point scale; *p* < 0.001), with significantly higher means in Attention, Confidence, Satisfaction, and Relevance (*p* < 0.05). Open-ended comments were more positive with AR (75.8% vs. 31.0%), while graphics-related complaints were more frequent with the Genially^®^ resource (34.5% vs. 75.0%). **Conclusions**: WebAR achieved higher RIMMS motivation scores than a content-matched interactive presentation. Adding 3D spatial interaction to otherwise equivalent materials can enhance learners’ motivation for caries detection training, while remaining low-cost and scalable.

## 1. Introduction

Dental caries remains the most prevalent chronic condition worldwide [1], and accurate diagnosis is a core competence in undergraduate dental education. In the last decade, criteria for defining, diagnosing, and making decisions regarding caries management have been modified and adapted to current scientific evidence [2,3]. The European Organization for Caries Research (ORCA) and the Association for Dental Education in Europe (ADEE) in their “European Core Curriculum in Cariology for undergraduate dental students” (ECCC) underscore the need for systematic training in risk assessment, caries detection, and decision-making using standardized frameworks such as ICDAS/ICCMS. These policies converge on the same objective: students must learn to integrate clinical and radiographic cues to stage lesions and plan minimally invasive care [4].

The emergence of artificial intelligence (AI) in dental diagnostics is expected to reshape the dental profession as we know it [5]. AI algorithms can detect carious lesions from periapical radiographs, bitewing radiographs, extraoral radiographs (such as panoramic or cone-beam computed tomography images) and intraoral photographs with satisfactory accuracy [6]. However, while such systems may assist in the detection of dental caries in clinical practice, diagnostic responsibility and clinical judgment will continue to rest with clinicians. Accordingly, sustaining students’ motivation to learn and practice caries diagnostic skills remains essential, perhaps even more so as AI gains popularity.

According to the ARCS Motivational Design model, motivation in learning is sustained through four dimensions: Attention, Relevance, Confidence, and Satisfaction (ARCS). To motivate students to learn, it is essential to stimulate their curiosity and interest in the subject (Attention), demonstrate the connection of the learning object with their personal goals (Relevance), and foster their confidence (Confidence). Moreover, to ensure a continuing desire for learning, students must feel satisfied with the results of the learning experience (Satisfaction) [7].

Technology-enhanced learning has expanded rapidly in dental education and is frequently associated with higher engagement and learner satisfaction. Among these tools, Augmented Reality (AR) and Virtual Reality (VR) are highlighted as relevant for the future of dental education [8,9]. AR is a technology that can superimpose digital information on the real world, combining reality and virtuality [10]. Some of the claimed positive effects of AR in education are: better learning outcomes, especially if the concepts are strongly related to spatial abilities; an increase in students’ motivation, satisfaction, interest, and focus; and better acquisition of motor skills [11,12,13,14].

A variety of hardware and software can be used to experience AR, from complex and high-cost equipment, such as head-mounted displays, to affordable mobile phones [15]. One of the simplest and cost-effective AR modalities is Web-based Augmented Reality (WebAR), which consists of an AR experience delivered through a standard web browser, allowing users to access it without installing an app [16]. The integration of WebAR and authoring tools, which allow users with basic computer knowledge to create applications using preprogrammed elements, is claimed to empower teachers to create their own educational content, democratizing the use of AR [17].

The contribution of AR-based learning to motivate students has been addressed in several fields, reporting promising results, boosting all ARCS dimensions compared to alternative educational resources. Notably, the majority of studies reviewed in a recent meta-analysis showed a significant increase in Attention among students who learned with AR [18]. Specifically, AR has been employed for educational purposes in Dentistry with promising results in various areas, including dental anatomy [19,20,21] head and neck anatomy [22], dental charting [23], oral histology [24], dental anesthesia [25], operative dentistry [26,27], and radiology [28]. Nevertheless, the application of AR to Cariology, particularly caries detection tasks, remains limited, and comparisons against other educational resources are still needed.

Therefore, this study aims to (i) design and document two isomorphic learning objects for caries detection—a WebAR experience and a content-matched interactive 2D presentation—built from authentic patient records; (ii) to compare their effect on third-year dental students’ motivation using the Reduced Instructional Materials Motivation Survey (RIMMS), with the RIMMS total as the primary outcome and the ARCS subscales (Attention, Relevance, Confidence, Satisfaction) as secondary outcomes; and (iii) to summarize open-ended feedback given by students after using the tools.

The null hypothesis (H0) states that WebAR does not differ from an interface-matched 2D presentation in students’ motivation (RIMMS total and ARCS subscales) for learning caries detection.

## 2. Materials and Methods

### 2.1. Ethical Committee Approval

The research protocol and the informed consent forms were revised and approved by the Research Ethics Committee of the Universitat de València (Spain) with registration number 1950552.

### 2.2. Pedagogical Rationale and Learning Objectives

The learning resources were created to support diagnostic reasoning in Cariology using authentic patient material, with tasks aligned to ICDAS/ICCMS learning objectives [29]. Although the tools enable practice in visual–radiographic caries detection, lesion staging, and minimally invasive decision-making, the study’s primary endpoint was students’ motivation rather than diagnostic performance.

This study was grounded in Keller’s ARCS model of motivational design. Both learning objects were designed to stimulate ARCS components under equivalent content and task demands: capturing Attention through interactivity; enhancing Relevance via authentic patient records and ICDAS-aligned tasks; fostering Confidence with clear goals and a simple interface; and eliciting Satisfaction by providing a coherent, enjoyable learning experience.

The learning experience followed an inquiry-based learning (IBL) approach [30], which is based in an active pursuit of knowledge proposing authentic activities to learners, giving them the chance to apply it. Rather than presenting theoretical content, the resources pose a challenge to students: explore authentic clinical records, apply ICDAS/ICCMS criteria to propose a diagnosis, and adopt a clinical decision.

### 2.3. Source Case

All assets used to design the learning resources were derived from a single, de-identified patient, who presented several carious lesions and existing restorations. Written informed consent was obtained for the use of clinical and radiographic records for educational purposes. Records obtained from the patient included 3D intraoral-scanner models of the maxillary and mandibular arches in Polygon File Format (PLY) format and tooth-level media (clinical photographs and periapical radiographs of teeth with clinically identifiable carious lesions), all anonymized for educational use. Digital impressions and intraoral images were acquired using the Medit i500 intraoral scanner (Medit Corp., Seoul, Republic of Korea).

### 2.4. WebAR Learning Object Development

The WebAR experience was developed in Zapworks Studio^®^ v6.5 (Zappar Limited, London, UK). As a preparatory step, 3D models obtained from the intraoral scanner underwent a refining and conversion process to meet the AR software requirements. First, using the manufacturer’s Medit i500 software (Medit Scan for Clinics v1.7), meshes were cleaned, obvious artifacts were removed, and bases were generated. The resulting PLY files were then imported into Blender^®^ v 2.92 (Blender Foundation, Amsterdam, The Netherlands) for optimization and texturing. Since the original PLY files contained vertex colors (not supported by Zapworks Studio^®^), UV maps were created, and vertex colors were baked into texture atlases. In addition, meshes were lightly decimated to reduce polygon count while preserving occlusal detail, and surface normals were checked and corrected to ensure a stable appearance across devices and to minimize file size. Optimized models were exported as OBJ/MTL (Wavefront 3D Object File/Wavefront Material Template Library) with their associated texture files. For distribution, the optimized 3D models and textures were uploaded to Sketchfab^®^ (Sketchfab, Inc., New York, NY, USA) (available online: https://sketchfab.com) to leverage fast web streaming and straightforward embedding within Zapworks Studio^®^ v6.5.

Additional assets imported into Zapworks Studio^®^ included clinical images and radiographs in JPEG format, and interface elements (labels, buttons and icons) in PNG format, which were designed in Microsoft PowerPoint^®^ (Office 2016) (Microsoft Corporation, Redmond, WA, USA).

Interactivity was implemented by creating tooth-specific hotspots on the 3D arch models. Tapping a hotspot opened the linked clinical and radiographic images. In total, 18 screens were developed, and interface elements were endowed with interactivity to enable navigation between screens.

An image-tracked experience was then configured so that a printed target acted as the marker, anchoring the 3D models and associated assets within the device’s camera view. The tracking target was designed in Canva^®^ (Canva, Perth, Australia) (available online: https://www.canva.com).

Finally, the AR experience was published to the web and made accessible via QR code or a short URL for easy sharing with students.

### 2.5. Interactive 2D Presentation

To test the WebAR resource’s ability to motivate students, a comparator was needed. Rather than selecting a conventional resource to compare with, we opted for a content- and interface-matched interactive 2D presentation as an active control. For this purpose, a Genially^®^ presentation (Genially Web SL, Córdoba, Spain) (available online: https://genially.com), was created to mirror the AR resource in content, interface and navigation while restricting visualization to 2D. The same patient assets (clinical photographs and radiographs) were used, with the exception of the 3D models, which were replaced by 2D images showing their frontal, left and right lateral and occlusal perspectives. Equivalent interactive tooth hotspots were included, which, when selected, displayed linked images and radiographs. The Genially^®^ resource was structured into an interactive slide sequence, each one equivalent to a screen of the WebAR experience.

This isomorphic design ensured that the planned contrast between conditions was limited to visualization mode (2D versus 3D/WebAR).

**Figure 1:**
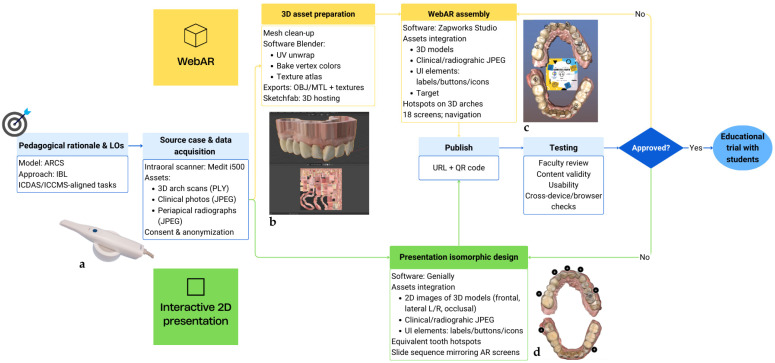
Design–build workflow for creating two isomorphic learning resources for caries detection training. A shared trunk (shown in blue) branches into parallel development tracks—the yellow WebAR track and the green Genially® interactive 2D presentation track—which re-merge for publication, faculty testing and final iteration before starting the educational trial. (**a**) Medit i500^®^ intraoral scanner used to capture the source case. (**b**) Blender^®^ scene showing UV-mapped model and texture atlas. (**c**) WebAR overlay anchored by an image-tracking target with hotspot icons. (**d**) Genially^®^ slide reproducing the same tooth hotspots in 2D. **Abbreviations:** ARCS—Attention, Relevance, Confidence, Satisfaction; IBL—Inquiry-Based Learning; ICDAS—International Caries Detection and Assessment System; ICCMS—International Caries Classification and Management System; WebAR—Web-based Augmented Reality; 2D—two-dimensional; 3D—three-dimensional; UI—user interface; URL—Uniform Resource Locator; QR—Quick Response code; OBJ/MTL—Wavefront object/material file formats; L/R—left/right.

Likewise, a short URL and a QR code were generated to share the resource with students. The entire resource creation flowchart is represented in Figure 1.

### 2.6. Iteration and Testing

The build process included internal walkthroughs with course faculty to verify content validity and usability (label clarity, hotspot placement and legibility of patient records). The compatibility of the resources across different devices and operating systems was also tested before sharing them with students. Any minor issues detected during this phase were addressed and corrected to ensure optimal user experience.

### 2.7. Study Design

A randomized controlled experimental study was planned to compare the impact of two interactive resources (WebAR vs. 2D presentation) on undergraduate dental students’ motivation to learn caries detection. The study took place at the Dentistry School at the Universitat de València as part of the Cariology course taught in the third year of the Dentistry degree program.

To determine the required sample size, a power analysis was conducted using the online Sample Size Calculator (version 1.063) developed by Robert Ristl, from the University of Vienna (https://homepage.univie.ac.at/robin.ristl/samplesize.php?subject=support; accessed on 4 January 2021). Based on the observed means and standard deviations for motivation scores in similar previous studies, a minimum of 22 participants per group was determined to be sufficient to achieve a power of 80% with a significance level of 0.05.

Recruitment was conducted among third-year dental students from the Universitat de València via the institutional learning management system (LMS). Interested students were provided with an overview of the study and invited to voluntarily participate. It was clarified that participation was voluntary, with no monetary or academic compensation offered to participants. Additionally, there were no negative consequences for non-participating students, who could also use the new tools if they wished. The inclusion criteria required participants to be enrolled in the Cariology course, and there were no exclusion criteria.

A total of 92 students were voluntarily recruited for participation. After written informed consent was obtained, students were randomly assigned to one of two groups: the WebAR group (AR) or the interactive 2D presentation group (G). To perform the random allocation into the two groups, an independent researcher assigned a number to each participant, and used the random number generator web tool developed by the University of Granada (Spain) (https://www.ugr.es/~jsalinas/Aleatorios.htm; accessed on 25 January 2021) to ensure an equal distribution.

### 2.8. Educational Intervention

The intervention was designed as a single-session learning activity. Participants were exposed to their assigned learning object in separate sessions.

At the start of each session, written instructions to access the assigned tool were given to all students. Participants used their own mobile devices to access the resource via a QR code; an internet connection was required. Students in the AR group were provided with a printed target to activate the image-tracking experience. Both resources included an info button that offered guidance on how to navigate the tool. Due to the simplicity of the interface, students did not receive additional training on the use of the resources.

All participants had 90 min to use the assigned tool and, individually and anonymously, completed the following tasks in order:

1. Specify the ICDAS II code for each tooth [29];
2. Specify the radiographic code following ICCMS [31] for observable teeth in the radiographs;
3. Propose the appropriate treatment for each tooth with a carious lesion;
4. Complete an adapted version of the Reduced Instructional Materials Motivation Survey (RIMMS).
5. The RIMMS is a 12-item instrument, a reduced form of the 36-item Instructional Materials Motivation Survey (IMMS). Both questionnaires are grounded on the ARCS motivational design model [32]. The 12 items can be grouped into four subscales: Attention, Relevance, Confidence and Satisfaction. Items are scored on a 5-point Likert scale, where 1 is considered the least favorable and 5 the most favorable. This instrument has been validated and it is acceptable for evaluating each one of the four dimensions [33]. The RIMMS was translated into Spanish, and terminology referring to instructional materials was adapted to better suit multimedia resources (Table 1).

Finally, an optional open-ended question invited students to provide written feedback on the resource they used.

### 2.9. Statistical Analysis

In this study, student motivation was the only educational outcome assessed. Although participants performed the clinical and radiographic diagnosis of the case, neither their diagnostic accuracy nor other potential learning outcomes, such as gains in theoretical knowledge or knowledge retention, were evaluated. Statistical tests were conducted using SPSS 28.0.0 software (IBM Corp., Armonk, NY, USA). Descriptive analysis included calculating the frequency, mean, standard deviation, median, and interquartile range for each item, for each subscale (Attention, Confidence, Satisfaction, and Relevance), and for the total RIMMS score. Subscale scores were calculated as the arithmetic mean of their three constituent items, yielding subscale scores on a 1–5 scale, with higher scores indicating greater motivation. The total RIMMS score was defined as the mean of the four subscale means and likewise ranged from 1 to 5. Internal consistency (Cronbach’s α) was estimated for each subscale and for the total; values ≥ 0.7 were considered acceptable. The Shapiro–Wilk test was applied to assess normality. Considering that data did not follow a normal distribution, non-parametric tests were used for subsequent analyses. Between-group differences in subscale and total scores were tested with the Mann–Whitney U test. Statistical significance was established if *p* < 0.05.

The qualitative analysis of participants’ open-ended responses was conducted using ATLAS.ti software, version 23 (ATLAS.ti Scientific Software Development GmbH, Berlin, Germany). Initially, a preliminary reading of all responses was performed to identify the main themes, and codes were created to represent each theme. Subsequently, a re-reading of the responses took place, which were classified under the appropriate codes, and code frequencies by group were calculated. Additionally, DATAVIV’s online tool (Le Sphinx Iberoamérica S.L., Madrid, Spain) was used to conduct a keyword-frequency analysis of the open-ended responses for each group.

## 3. Results

### 3.1. Developed Learning Objects

Both resources are accessible via QR code or URL on any device with an internet connection, regardless of browser or operative system, and free of charge. To use the WebAR experience, the device must also have a camera. In addition, a physical target is required (Supplementary Figure S1), which can be printed in color or grayscale.

**Figure 2:**
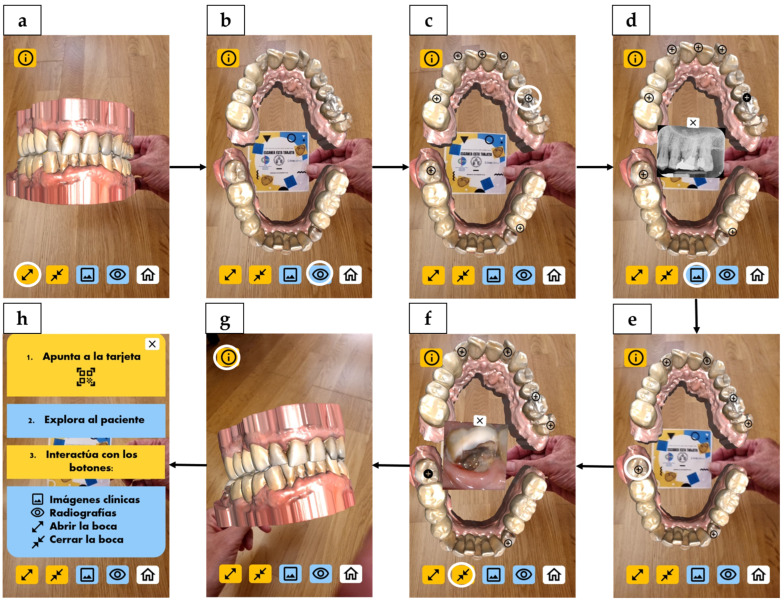
WebAR learning object—representative screens and interactions. Arrows indicate navigation paths between screens after activating the corresponding buttons (circled in white). (**a**) “Home” screen, with a bottom toolbar and an info button at the top left, both present in every screen. Three-dimensional models (in occlusion by default) are anchored to the printed target (image-tracking experience); (**b**) “Open mouth” screen; (**c**) “Radiographic images” screen, with tooth-level hotspots displayed across the arches; (**d**) Periapical radiograph opened in a pop-up from a hotspot (close button visible); (**e**) “Clinical images” screen, with tooth-level hotspots; (**f**) Pop-up displaying a clinical photograph opened from a hotspot; (**g**) “Closed mouth” screen; (**h**) Instruction overlay (Spanish) with step-by-step guidance (“1. Aim at the target; 2. Explore the patient; 3. Interact with the buttons”) and a legend for the icons (top to bottom: clinical images, radiographs, open mouth, close mouth).

WebAR resource is available at: https://webxr.run/Wo0R9enmvJ3Mm (accessed on 1 September 2025). After tapping “Launch”, the “Home” screen opens. A bottom toolbar provides five controls (left to right): open mouth (disocclude), close mouth (occlude), clinical images, radiographic images and “Home”. When the device’s camera is aimed at the printed target, the patient’s 3D models appear anchored to it within the WebAR scene. They respond to the selected on-screen controls, transitioning from occlusion to disocclusion. Users can rotate and zoom the 3D models by moving the physical target or the device, or by using standard touch gestures (drag/pinch). On the “Clinical images” and “Radiographic images” screens, the arches are shown disoccluded and tooth-level hotspots appear; tapping a hotspot opens the corresponding image. An info button remains visible at the top left and opens contextual help. Holding the device in portrait orientation throughout the experience is recommended. Figure 2 displays an overview of the WebAR resource.

**Figure 3:**
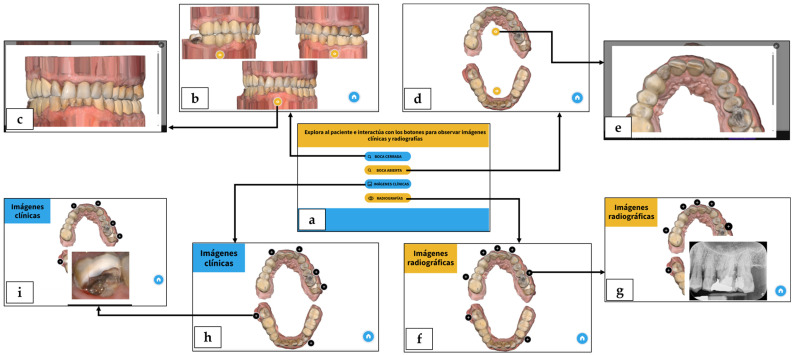
Overview of the Genially^®^ 2D interactive presentation. Arrows indicate navigation paths between slides after activating the corresponding buttons. (**a**) “Home” slide with instructions in Spanish (“Explore the patient and interact with the buttons to view clinical and radiographic images”) and four navigation buttons; (**b**) “Closed mouth” slide; (**c**) Close-up of the arches in occlusion; (**d**) “Open mouth” slide; (**e**) Close-up of an arch in occlusal view; (**f**) “Radiographic images” slide with tooth-level hotspots; (**g**) Pop-up of a periapical radiograph after hotspot activation; (**h**) “Clinical images” slide with tooth-level hotspots; (**i**) Pop-up of a clinical image after hotspot activation.

The Genially^®^ 2D interactive presentation is available at: https://view.genial.ly/61d47a2b019bb20de6e64f17/interactive-content-paciente-diagnostico (accessed on 1 September 2025). Upon access, the “Home” slide appears with instructions and four navigation buttons (top to bottom): closed mouth (occlude), open mouth (disocclude), clinical images, radiographic images. Selecting a button opens the corresponding slide. On the “Closed mouth” slide, 2D images of the models in occlusion (left lateral, right lateral, and frontal) are shown, each with a “Zoom” button; selecting it opens a close-up of the same image. Rotation is not available. On the “Open mouth” slide, 2D occlusal views of the upper and lower arches are displayed, also with “Zoom” buttons. On the “Clinical images” and “Radiographic images” slides, the same occlusal views include tooth-level hotspots; hovering over a hotspot opens the corresponding image. A “Home” button remains visible at the bottom right to return to the main menu. Holding the device in landscape orientation throughout the experience is recommended. Figure 3 displays an overview of the Genially^®^ resource.

### 3.2. RIMMS Results

Of the initial sample of 92 students, 85 ultimately participated in the educational experience (92.4% of the target population): 46 used the AR tool (AR group) and 39 used the interactive presentation (G group). Reasons for withdrawal were schedule conflicts or unannounced non-attendance. Participants were 30.6% female and 69.4% male, with a mean (SD) age of 22.44 (3.95) years (range 20–45).

Internal consistency was high in both groups (Cronbach’s α = 0.92 for AR; α = 0.89 for G), as shown in Table 2.

AR group achieved a higher RIMMS total score (mean ± SD = 4.14 ± 0.70, median = 4.25, IQR = 1.17) than the G group (mean ± SD = 3.53 ± 0.71, median = 3.58, IQR = 1.13). Mann–Whitney U test showed statistically significant differences between groups (U= 488, *p* < 0.001). Additionally, AR group rated every item of the survey higher, as shown in Table 3.

In the Attention dimension, the AR group’s highest rating was for item Q2 (4.35 ± 0.74), while the G group’s highest was Q1 (3.67 ± 0.96). In Confidence, the highest-rated item was Q6 for the AR group (3.76 ± 1.12) and Q4 for the G group (3.36 ± 1.11). For Satisfaction, both groups rated Q9 highest (AR 4.35 ± 0.90; G 3.51 ± 1.14). Finally, in the Relevance dimension, the AR group provided the highest score for item Q11 “The content and presentation of the resource convey the impression that knowing how to do the task is worthy” (4.37 ± 0.77), whereas the G group did so for Q10 “It is clear to me how the content of this material is related to things I already know” (4.15 ± 0.78). These were the highest-rated items across the survey for their respective groups.

Mann–Whitney U tests comparing the AR and G groups showed statistically significant differences across all four subscales and for the total score (Table 4), as noted above, rejecting the null hypothesis. In both groups, Relevance was the highest-rated subscale, whereas Confidence received the lowest ratings.

**Figure 4:**
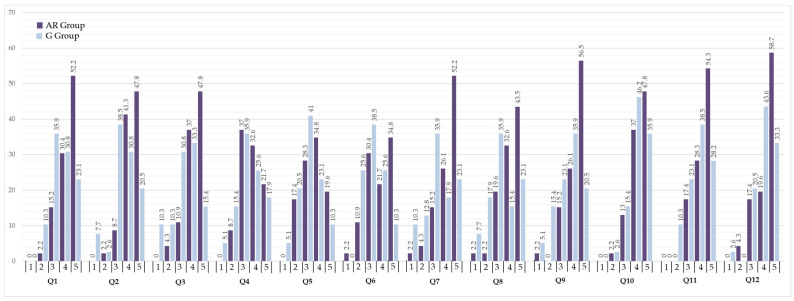
RIMMS response percentages by item and group (AR Group = dark purple; G Group = light blue). For each item (Q1–Q12), bars show the percentage of students selecting that option (1–5; least to most favorable).

The number of participants who rated RIMMS items with 4 or 5 was higher in the AR group than in the G group, as shown in Figure 4.

### 3.3. Qualitative Analysis of Open-Ended Responses

63% of the students in the AR group and 41% of the students in the G group responded to the voluntary open-ended question about the resource they used.

Thematic analysis identified the following main themes: (i) positive appraisal of the resource; (ii) suggestions for improvement or reports of issues related to the number/quality of images or usability; and (iii) complaints about the difficulty of the diagnostic task. Exclusively in the AR group, a theme related to the didactic potential of the tool was identified, and in the G group, there was a demand for a three-dimensional view. Table 5 shows theme frequencies by group. Some responses were coded under more than one theme.

A larger number of AR users expressed positive adjectives or feelings about the tool or the activity (75.8% compared to 31.0% in the G group). Conversely, a higher proportion of G responses mentioned issues with image quality/quantity (75.0% vs. 34.5% in AR). One G student also suggested including the solution to the diagnostic challenge along with an explanation.

**Figure 5:**
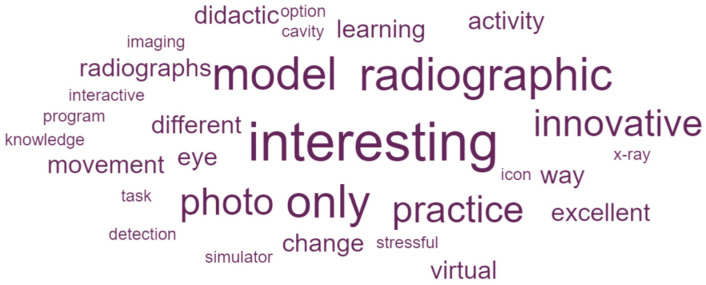
Word clouds visualizing term frequencies for AR group open-ended responses. The larger the word, the higher the frequency.

**Figure 6:**
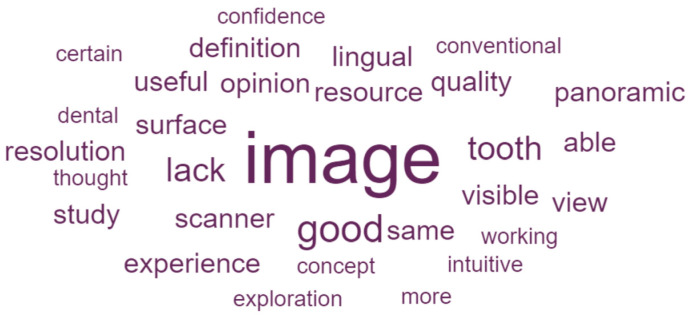
Word clouds visualizing term frequencies for G group open-ended responses. The larger the word, the higher the frequency.

As a result of the keyword-frequency analysis of the open-ended responses, word clouds for AR group (Figure 5) and G group (Figure 6) were created. In the AR group, prominent terms such as *interesting*, *innovative*, *model*, *radiographic*, and *photo* point to a positive appraisal of the spatial 3D experience, and reference to the available case assets (e.g., “An interesting resource for becoming familiar with the technology and with this course”; “The image quality could be improved a bit, but I liked it a lot and I think this can help us in practice and in learning”; “Very instructional and encouraging for learning”). In contrast, the G group cloud is dominated by *image*, co-occurring with *quality*, *definition*, *resolution*, and *lack*, alongside requests for additional viewing options, such as *lingual* or *panoramic*) (e.g., “Few images and low image quality. It would be better to be able to view several at the same time”; “In my opinion, there were missing clinical and radiographic images of the teeth; there were images, but several tooth surfaces were not shown”).

## 4. Discussion

### 4.1. Key Findings

Two interactive resources for learning caries detection were developed with simple, no-cost authoring tools by faculty to address specific educational needs. Both tools were freely accessible and designed for zero-install, browser-based access via QR/URL on common smartphones, tablets, and laptops. The main difference between the resources was visualization: the WebAR object enabled spatial exploration of authentic 3D dental arches, whereas the Genially^®^ resource presented equivalent content in 2D.

Results indicated that both resources positively affected students’ motivation. However, WebAR was more favorable for motivating students in learning caries detection methods than the 2D interactive presentation, as significantly higher ratings were obtained on the RIMMS total and on all four ARCS subscales (Attention, Relevance, Confidence, Satisfaction). Thus, the null hypothesis was rejected.

In both groups, the dimension with the highest score was Relevance, indicating that these tools are perceived as useful for students. Conversely, Confidence was lowest in both groups. Taken together, these patterns suggest that adding spatial interaction to an otherwise equivalent interface/content is associated with greater perceived motivation, particularly for features tied to attention capture and perceived usefulness of the activity for clinical learning.

### 4.2. Comparison with Similar Studies

Our findings are consistent with prior work examining dental students’ attitudes toward AR technologies. To our knowledge, this is the first study to focus primarily on dental students’ motivation with AR; nevertheless, several studies have examined conceptually related ARCS dimensions—Attention, Relevance, Confidence, and Satisfaction—often under different labels. Only one study employed the IMMS, comparing an AR serious game about osteoclasts with text-based learning, although knowledge gain was the primary aim. It was found that learning outcomes were comparable to text-based self-learning, but the AR serious game motivated students with significant differences in Confidence, Satisfaction and Relevance compared to the non-AR control [34].

The other related studies assessed at least one of the ARCS dimensions using specifically developed questionnaires by own authors, making it challenging to compare across different studies. Nevertheless, we can extract similarities between these and our study. In a study by Mladenovic et al., 63.2% of students agreed or strongly agreed that their confidence in administering anesthesia had increased after using the AR resource, aimed at teaching local anesthesia administration. However, this question received lower scores compared to others, aligning with our results. 89.4% of students agreed or strongly agreed that they felt engaged with the activity [35], and in another study evaluating the same tool, this value ranged between 78% and 88% [25]. These questions could be related to the Attention dimension, which also received high ratings in the present study. Additionally, 89.9% of students agreed or strongly agreed that the resource was helpful for learning [34], and Zafar et al. obtained a similar percentage for the same question [25]. These questions, related to the Relevance of the tool, received the highest ratings from students, consistent with the results of our study.

In another study focused on the learning of dental radiology, the impact on students’ perceptions of an AR resource and a traditional textbook was compared. Students who used AR reported higher levels of confidence, attention, and satisfaction, with statistically significant differences. Consistent with our results, the values assigned to questions related to Confidence received lower scores than other variables [36].

The use of new technologies may entail technical issues that can negatively affect students’ perceptions. In our study, 20.7% of comments from AR users mentioned usability problems or suggestions for improvement. When compared to other experiences, a study on the use of AR for dental anatomy learning reported that 50% of students did not find it easy to use [19]. In another study using the HoloHuman^®^ app for head and neck anatomy learning, only 36.5% of students agreed it was easy to use [22]. Similar findings were obtained by Mahrous et al., with only 18% of students considering the AR resource easy to use compared to natural and 3D-printed teeth [37]. Conversely, Sharmin et al. found that 89% of students rated their AR application for oral histology learning as very easy to use, although the sample size was limited [24]. We consider that, in our case, the reported usability issues did not have a substantial negative impact on student motivation or satisfaction. In the G group, fewer usability issues were reported (13% of comments), yet motivation scores were lower than in the AR group.

Focusing on methodological aspects, it is noteworthy that both the 3D models and the learning tools were developed specifically for this educational experience, consistent with most AR reports in dentistry [19,26,27,38,39,40]. This may be attributed to the absence of commercially available applications that match the researchers’ objectives.

The choice of image-tracking technology, with the use of markers and mobile devices to visualize the AR experience, also aligns with the methodology followed by most recent studies in the field [39,40], and the majority of the previously cited literature. However, an AR experience based on object tracking has recently been developed for wax-up training: it dispenses with markers and may improve usability, although it still needs to be investigated [41].

In contrast to the widespread use of mobile AR, other authors have employed head-mounted displays, such as HoloLens^®^ glasses or other devices [20,22,42]. These devices were excluded from the present study because of cost constraints and reported adverse effects, including nausea, headache, or blurred vision, as highlighted by Zafar and Zachar [22].

Regarding the complexity of the AR resource, most studies report using professional software (e.g., Unity/Vuforia; ARKit/ARCore) to develop native apps [39]. Typically, this development relies on collaborations between dental schools and other university departments, such as Engineering or Computer Science [34,43,44,45], or with external companies [46,47]. Less commonly, dental educators themselves possess the necessary programming or 3D design skills [41,48], whereas the use of browser-delivered WebAR and authoring tools, as in our study, remains scarcely documented in peer-reviewed dental education literature, with only one other example reported [49]. WebAR provides advantages over native apps, which may constrain scalability due to installation requirements, device compatibility, licensing, and maintenance burdens, whereas our approach lowers implementation costs, as all software used was cost-free, and addresses barriers highlighted in the literature [50]. Moreover, WebAR facilitates easy sharing of educational resources, extending its impact beyond students to the broader scientific and educational community, fostering transparency, reproducibility of educational interventions, and collaborative refinement of the tool across different curricular contexts.

### 4.3. Study Limitations

This study has several limitations. First, it was conducted at a single institution with one cohort and a sample size constrained by course enrollment, which may limit generalizability. However, sample sizes in studies similar to ours are comparable or even smaller, ranging from 9 participants [24] to 217 [36].

Second, blinding of participants and instructors was not feasible; although we used a content- and interface-matched 2D comparator to mitigate a novelty effect, expectancy or modality preferences cannot be fully ruled out. In trials of digital and behavioral interventions where blinding is infeasible, active controls are recommended to balance non-specific factors [51]. By exposing both groups to equally novel, interaction-rich digital resources, we aimed to reduce novelty/Hawthorne effects.

Third, each student used their own mobile device, introducing uncontrolled heterogeneity that may alter the experience. The tools may function differently depending on the device used, and the impact of these factors on student motivation was not studied. Additionally, no specific calibration or training on the use of the tools was provided, as their usability was considered simple and intuitive, and students were expected to be familiar with the operation of their own devices. However, some usability complaints were reported by students in both groups. Providing prior training might improve the educational experience and enhance the validity and reliability of the study.

Finally, qualitative comments were optional, with unequal response rates between groups and coding focused on frequencies, so response bias is possible.

The study’s primary outcome was self-reported motivation (RIMMS) collected immediately after a single session; we did not measure diagnostic accuracy, longer-term retention, or transfer to clinical performance. We therefore acknowledge the absence of demonstrated instructional effectiveness as a key limitation. Nonetheless, WebAR’s potential to enhance motivation should not be undermined. Evidence from dental education and broader higher-education contexts indicates that higher motivation is associated with better academic performance [52,53,54]. Crucially, the quality of motivation appears decisive: autonomously motivated students are more likely to adopt deep learning approaches that correlate positively with concurrent performance, whereas controlled motivation and amotivation are linked to surface approaches and poorer outcomes [54]. These findings suggest that enhancing autonomous motivation is not merely about engagement but plausibly part of the causal pathway to better learning, providing a theoretically grounded rationale for targeting motivation in educational innovations.

At the same time, we should be aware of recent concerns about the negative effects of technology overuse on student performance. Digital overexposure without instructional coherence causes academic underperformance, but pedagogically founded and optimally dosed instructional tools can create a positive and engaging learning environment [55]. Accordingly, AR and other technology-enhanced resources should be implemented only when pedagogically justified and when they demonstrably address limitations of conventional approaches (e.g., constraints of space, time, or material resources). Future studies should pair AR with principled instructional design and include objective learning endpoints to delineate its educational value. As next steps, we will refine the WebAR resource by improving image resolution and expanding the case library by adding new patients. Following students’ suggestions, we plan to add instructional feedback to bolster their confidence. With continued refinement and rigorous evaluation, WebAR can evolve into a scalable learning resource that brings authentic cases to dental students’ own devices—turning passive study into active, sustainable, clinic-ready practice.

## 5. Conclusions

In this single-session, randomized study, a WebAR learning resource, built from authentic patient records and accessible on students’ own devices, was associated with higher motivation than an interface-matched 2D presentation for the RIMMS total and all ARCS subscales. These findings suggest that adding spatial interaction to otherwise equivalent materials can enhance learners’ motivation for caries detection training, while remaining low-cost and scalable.

## Figures and Tables

**Table 1:** Adapted version of the Reduced Instructional Materials Motivation Survey (RIMMS) used in this study. Items are grouped by the ARCS subscales—Attention, Relevance, Confidence, Satisfaction—and were minimally reworded to refer to multimedia resources.

Item	Question	Dimension
Q1	The quality of the resources helped to hold my attention.	Attention
Q2	The way the information is presented on the resources helped keep my attention.	
Q3	The variety of interactive material helped keep my attention on the task.	
Q4	As I worked with the resource, I was confident that I could successfully complete the task.	Confidence
Q5	After working with the resource for a while, I was confident that I would be able to do the same task on a real patient.	
Q6	The good representation of the contents helped me be confident that I would learn to do the task.	
Q7	I enjoyed this resource so much that I would like to use more similar resources.	Satisfaction
Q8	I really enjoyed using this resource.	
Q9	It was a pleasure to work with such a well-designed resource for the task I had to solve.	
Q10	It is clear to me how the content of this material is related to things I already know.	Relevance
Q11	The content and presentation of the resource convey the impression that knowing how to do the task is worthwhile.	
Q12	The knowledge obtained by using this resource will be useful to me.	

**Table 2:** Internal consistency (Cronbach’s α) for the RIMMS subscales and total score by group. All coefficients indicated high reliability.

	Cronbach’s α	
Group	AR	G
Attention	0.91	0.88
Confidence	0.91	0.89
Satisfaction	0.91	0.88
Relevance	0.91	0.88
Total	0.91	0.88

**Table 5:** Qualitative analysis of open-ended responses from AR and G groups. The codes with their description, their frequency in number (N) and percentage (%), and an example for each one of them are provided.

Code	Description	Frequency (N/%)		Example Phrases
		AR	G	
Positive appreciation of the resource	Includes any word or expression implying a feeling of satisfaction with the use of the tool, such as “interesting,” “innovative,” “excellent,” or “I loved it.”	22/75.8%	5/31%	“I found it an excellent teaching material. The most innovative activity throughout the entire course and its implementation is much needed. I really liked it!!” (AR) “Good tool for clinical practice.” (AR) “I really liked having access to this tool before engaging in patient practices, as I feel that I won’t be overwhelmed in a real clinical setting with some prior experience. I would greatly enjoy using this resource at home.” (G) “In my opinion it is a very good working tool although it does not give me the same security as conventional exploration.” (G)
Didactic tool	Mention is made of the utility of the resource as a learning tool, including words or expressions such as “didactic,” “learning,” or “knowledge.”	11/37.9%	0/0%	“I really liked this interactive program as it’s another way to learn and acquire the knowledge we need.” (AR) “I consider that it is useful as a complement in teaching and in patient practices to see cases, but that it could not replace them.” (AR) “I would have liked there to be more clinical photos, but I found it a very interesting resource from which I think you can learn a lot.” (AR)
Suggestions for improvement: Images	Comments demand a greater quantity or quality of clinical or radiographic images.	10/34.5%	12/75%	“I would have liked to see more clinical and radiographic images included.” (AR) “Good experience. Include more clinical and radiographic images.” (AR) “Lack of image resolution in the scan as well as more clinical images (photographs) and a panoramic radiograph.” (G) “Better image definition would be better.” (G)
Suggestions for improvement: Usability	Any suggestion for improvement or inconvenience related to usability, handling, navigation, or design of the tool is expressed.	6/20.7%	2/13%	“The idea needs better development. The graphics are lacking. Interaction is difficult; improve virtual navigation.” (AR) “The only thing I would change is to make the paper on which the model is placed more rigid. A plastic or cardboard surface would help prevent the model from disappearing and make it easier to move.” (AR) “The website hasn’t provided a very good experience, but the provided images have been very useful.” (G)
Suggestions for improvement: Demand for a three-dimensional view	Demand for the tool to include options to rotate or enlarge models, or to visualize all tooth surfaces.	0/0%	4/25%	“Lack of 3D rotation of the scanner view, more clinical images, and more radiographs. A panoramic radiograph for an overall view, increased scanner resolution. Improve zoom.” (G) “We do not see all the surfaces of the teeth to be studied, which limits diagnosis and study” (G) “Lack of definition in the scanner images. It’s a useful resource, but visualization would be easier if we could rotate the images”. (G)
Difficulty in diagnosis	Feelings related to insecurity or complexity in solving the requested diagnostic task are expressed.	5/17.2%	3/19%	“Quite educational, but there’s confusion in diagnosing some pathologies and treatments.” (AR) “Excellent tool for the student; perhaps diagnosis is made more difficult by not having a clinical view of the patient and relying only on images, but it is indeed ideal as a complementary method.” (AR) “Perhaps too many quadrants to work on and number.” (AR) “The image quality makes it difficult to see lesions clearly and evaluate them. Additionally, the palatal/lingual aspect wasn’t well visible.” (G) “I would add more clinical images, since scanned (three-dimensional) images alone are not enough to establish the diagnosis.” (G)

**Table 3:** Descriptive results for each RIMMS item (1–5; higher values indicate greater motivation) by group (AR vs. G), including mean, standard deviation (SD), median and interquartile range (IQR).

Dimension	Item	Mean ± SD		Median		IQR	
		AR	G	AR	G	AR	G
Attention	Q1	4.33 ± 0.82	3.67 ± 0.96	5	4	1	1
	Q2	4.35 ± 0.74	3.54 ± 1.10	4	4	1	1
	Q3	4.28 ± 0.83	3.33 ± 1.18	4	3	1	1
Confidence	Q4	3.67 ± 0.92	3.36 ± 1.11	4	3	1	1
	Q5	3.57 ± 1.00	3.13 ± 1.03	4	3	1	1.5
	Q6	3.76 ± 1.12	3.21 ± 0.95	4	3	2	1.5
Satisfaction	Q7	4.22 ± 1.01	3.31 ± 1.26	5	3	1	1
	Q8	4.13 ± 0.96	3.28 ± 1.23	4	3	1	1.5
	Q9	4.35 ± 0.90	3.51 ± 1.14	5	4	1	1
Relevance	Q10	4.30 ± 0.90	4.15 ± 0.78	4	4	1	1
	Q11	4.37 ± 0.77	3.85 ± 0.96	5	4	1	2
	Q12	4.33 ± 0.92	4.05 ± 0.89	5	4	1	1

**Table 4:** Mann–Whitney U test results comparing motivation between groups (AR vs. G) for each RIMMS subscale and the total score, including mean, standard deviation (SD), median, interquartile range (IQR), U statistic, Z statistic, and *p*-value.

Dimension	Mean ± SD		Median		IQR		U	Z	*p*
	AR	G	AR	G	AR	G			
Attention	4.32 ± 0.72	3.51 ± 0.93	4.33	3.67	1	1	454	−4.09	<0.001 *
Confidence	3.67 ± 0.84	3.23 ± 0.77	3.67	3.33	1.33	0.67	658	−2.29	0.022 *
Satisfaction	4.23 ± 0.87	3.37 ± 1.11	4.67	3.33	1.33	1	474	−3.91	<0.001 *
Relevance	4.33 ± 0.66	4.02 ± 0.69	4.5	4	1.25	0.83	683	−2.08	0.037 *
Total	4.14 ± 0.70	3.53 ± 0.71	4.25	3.58	1.17	1.13	488	−3.74	<0.001 *

* Statistically significant differences.

## Data Availability

The raw data supporting the conclusions of this article will be made available by the authors on request.

## Supplementary Materials

The following supporting information can be downloaded at: https://www.mdpi.com/article/10.3390/dj14010001/s1, Figure S1: WebAR target.

## Abbreviations

The following abbreviations are used in this manuscript:

2D	Two-dimensional
3D	Three-dimensional
ADEE	Association for Dental Education in Europe
AI	Artificial intelligence
AR	Augmented Reality
ARCS	Attention–Relevance–Confidence–Satisfaction (Keller’s motivational model)
ECCC	European Core Curriculum in Cariology
G	Genially^®^ group
ICCMS	International Caries Classification and Management System
ICDAS	International Caries Detection and Assessment System
IMMS	Instructional Materials Motivation Survey
IQR	Interquartile range
LMS	Learning management system
MTL	Material Template Library (Wavefront)
OBJ	Wavefront 3D object file format
ORCA	European Organization for Caries Research
PLY	Polygon File Format
PNG	Portable Network Graphics
QR	Quick Response (code)
RIMMS	Reduced Instructional Materials Motivation Survey
SD	Standard deviation
URL	Uniform Resource Locator
VR	Virtual Reality
WebAR	Web-based Augmented Reality
