# Supplementary figures and images for: Web-Based Augmented Reality vs. Interactive Presentation for Learning Caries Detection: A Randomized Study on Student Motivation
Source: Dent J (Basel). 2025 Dec 19;14(1):1. doi: 10.3390/dj14010001 (PMC12840382; doi:10.3390/dj14010001)

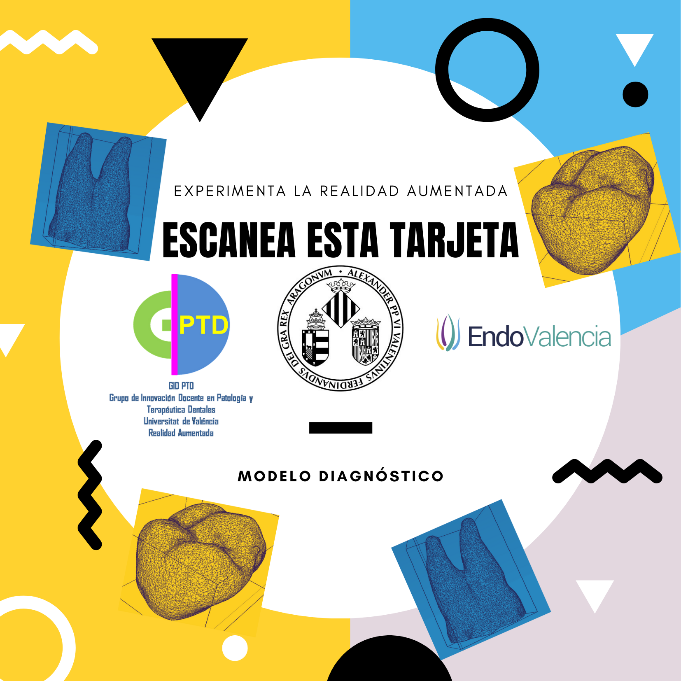

Supplement: Supplementary file 1 [file dentistry-14-00001-s001.zip › dentistry-3893949-supplementary.png]
